# Edaravone dexborneol compared to edaravone in the treatment of acute cerebral infarction: A meta-analysis

**DOI:** 10.3389/fphar.2025.1579742

**Published:** 2025-06-04

**Authors:** Yanshuo Shi, Yuanyuan Yue, Mi Wang, Huizhen Wu

**Affiliations:** ^1^ Department of Pharmacy, Hebei General Hospital, Shijiazhuang, China; ^2^ Hebei Key Laboratory of Clinical Pharmacy, Shijiazhuang, Hebei Province, China; ^3^ Hebei University of Chinese Medicine, Shijiazhuang, Hebei Province, China

**Keywords:** PubMed, Cochrane Library, Embase, CBM, CNKI, Wanfang, edaravone dexborneol, acute cerebral infarction, clinical efficacy

## Abstract

**Objective:**

The objective of this study was to systematically assess the clinical efficacy and safety of edaravone dexborneol compared to those of edaravone in treating acute cerebral infarction.

**Methods:**

We searched the PubMed, Cochrane Library, Embase, CBM, CNKI, Wanfang Database, and VIP to gather randomized controlled trials (RCTs) comparing edaravone dexborneol with edaravone for treating acute cerebral infarction, covering studies from the database inception to February 2024. After data extraction and quality evaluation, a meta-analysis was carried out using RevMan 5.3 and Stada 18.0 statistical software.

**Results:**

Seventeen RCTs were enrolled, including 2,778 patients, of which 1,493 and 1,285 were in the observation and control groups, respectively. The meta-analysis revealed that the total effective rate was significantly higher in the edaravone dexborneol group (RR = 1.17, 95% CI [1.11, 1.24], p < 0.00001) than in the edaravone group. Additionally, the rate of adverse reactions was significantly lower in the edaravone group (RR = 0.55, 95% CI [0.36, 0.82], p = 0.004). Fourteen days after treatment, the edaravone dexborneol group showed significantly better scores than the edaravone group in the NIHSS (MD = −2.13, 95% CI [-2.90, -1.35], p < 0.00001), Barthel Index (MD = 12.13, 95% CI [7.68, 16.58], p < 0.00001), and modified Rankin Scale (MD = −1.16, 95% CI [-1.75, -0.56], p = 0.0001).

**Conclusion:**

Edaravone dexborneol demonstrates superior clinical efficacy and safety compared to edaravone in the treatment of acute cerebral infarction, suggesting it may be a more effective therapeutic option.

## 1 Introduction

Acute cerebral infarction is a neurological disorder caused by the acute occlusion of cerebral arteries, leading to interrupted blood supply and resulting in functional deficits. The core pathophysiology involves oxidative stress, inflammatory cascade responses, and neuronal apoptosis triggered by the interruption of local cerebral blood flow in the ischemic area (Segura et al., 2008). Clinical manifestations of acute cerebral infarction include hemiplegia, speech disorders, and consciousness disturbances, severely affecting the quality of life of patients. According to the “China Stroke Prevention and Treatment Report (2023),” the number of stroke cases among individuals aged 40 years and older in China has reached 12.42 million, with over 2.8 million new cases of acute cerebral infarction each year, a disability rate of 75%, and direct medical costs exceeding 40 billion yuan, making it the leading cause of death and disability among adults (Segura et al., 2008). The main clinical treatment methods for acute cerebral infarction include intravenous thrombolysis, neuroprotective agents, anticoagulation, and mechanical thrombectomy. Although intravenous thrombolysis (such as alteplase) and endovascular mechanical thrombectomy have been established as the gold standard therapies for the acute phase, only approximately 10%–15% of patients can receive such reperfusion treatments in clinical practice due to strict treatment time windows (thrombolysis ≤4.5 h and thrombectomy ≤24 h), the risk of hemorrhagic transformation, and contraindications in some patients. For those who miss the optimal intervention window, neuroprotective treatment becomes a key strategy to delay secondary damage (Nagata, 2023; Berge et al., 2021). Edaravone, as a free radical scavenger, can exert some neuroprotective effects by inhibiting lipid peroxidation; however, its single-target action mode and limited blood–brain barrier penetration lead to individual differences in clinical efficacy (Watanabe et al., 2004). In recent years, the compound formulation of edaravone and dexborneol has shown synergistic enhancement potential in preclinical studies by combining the free radical scavenging effect of edaravone with the anti-inflammatory and blood–brain barrier-regulating dual mechanisms of dexcamphorol. Although several single-center studies suggest that it may improve neurological functional outcomes, there is currently a lack of large-sample, multicenter randomized controlled trial (RCT) data (Xu, et al., 2024), necessitating systematic evidence evaluation to clarify the clinical positioning and application value of this drug. In this study, we aim to integrate existing clinical evidence to provide an evidence-based decision-making basis for optimizing neuroprotective treatment pathways.

## 2 Materials and methods

This study was conducted in accordance with the Preferred Reporting Items for Systematic Reviews and Meta-Analyses (PRISMA) guidelines (Page et al., 2021). Ethical approval and consent were not required because all analyses were based on previously published studies.

### 2.1 Search strategy

We searched PubMed, Cochrane Library, Embase, CNKI, Wanfang, CBM, and VIP databases (search period from the database construction to Feb. 2024). The English search term included the following: “edaravone dexborneol” and “edaravone” and “acute cerebral infarction” or “ACI” or “cerebral infarction” and “randomized controlled trial” or “RCT.” Supplementary Material S1 provides detailed search strategies. The Chinese search terms were the Chinese forms of the above words.

### 2.2 Inclusion and exclusion criteria

#### 2.2.1 Exclusion criteria

The study excluded several categories: republished literature, reviews, and conference papers; studies with a sample size of fewer than 20 patients; those involving patients who had undergone anticoagulation and thrombolytic therapy before admission; and cases where full-text data could not be collected.

#### 2.2.2 Inclusion criteria

According to the PICOs principle, the following diagnostic criteria for acute ischemic stroke (ACI) were established based on the Chinese Guidelines for the Diagnosis and Treatment of Acute Ischemic Stroke (2018) (Peng et al., 2018): (1) acute onset, (2) focal neurological impairment with minimal total neurological impairment, (3) imaging showing responsible lesions/signs for at least 24 h, (4) nonvascular causes are excluded, and (5) cerebral CT or MRI ruled out cerebral hemorrhage. Research participants included patients aged from 18 to 80 years who met the diagnostic criteria for ACI, which was confirmed by CT or MRI. Interventions: the control group was treated with edaravone, and the experimental group was treated with edaravone dexborneol.

1. Outcome indicator: the degree of neurological impairment was assessed based on the National Institutes of Health Stroke Scale (NIHSS) (Abecassis et al., 2023). ② Self-care ability in daily life was evaluated using the Barthel Index (BI) (Yang, et al., 2022). Overall living ability was assessed using the modified Rankin Scale (mRS) (Tornero-Quiñones et al., 2020). ④ Clinical efficacy: total effective rate = (number of cured cases + number of apparent cases + number of effective cases)/total cases. The incidence of adverse reactions was used to evaluate the safety indices.
2. Research contents: papers published in any language of RCTs of edaravone dexborneol versus edaravone in the treatment of ACI.

### 2.3 Data extraction and quality evaluation

Two investigators independently screened the literature and determined whether they were included in this study. Data extraction information includes the title, author, object, method, measure, outcome, blind method, and allocation concealment.

Assessments were conducted by two investigators according to the Cochrane bias risk assessment tool. The assessment contents include the random sequence method, allocation concealment, blind method, data integrity, and other biases. The quality of the methodology was evaluated by two people separately, and different opinions were jointly judged by a third person.

### 2.4 Statistical method

Statistical analysis was performed using the RevMan 5.3 and Stada 18.0 software packages. The included data were represented by the relative risk (RR) and 95% confidence interval (CI). If there was no statistical difference in heterogeneity among the studies (I^2^ ≤ 50%, p ≥ 0.01), the fixed-effects model was used for analysis. If there was a statistical difference in heterogeneity among the studies (I^2^>50%, p < 0.01), the random-effects model was used for analysis (Zhu et al., 2023; Li et al., 2023). The mean difference (MD) was used as an effect analysis statistic for the continuous variables. In this study, a forest map was used to identify the analysis results of the data, and an inverted funnel map was used to represent the public offset results (Shi et al., 2019).

## 3 Results

### 3.1 General information

**FIGURE 1:**
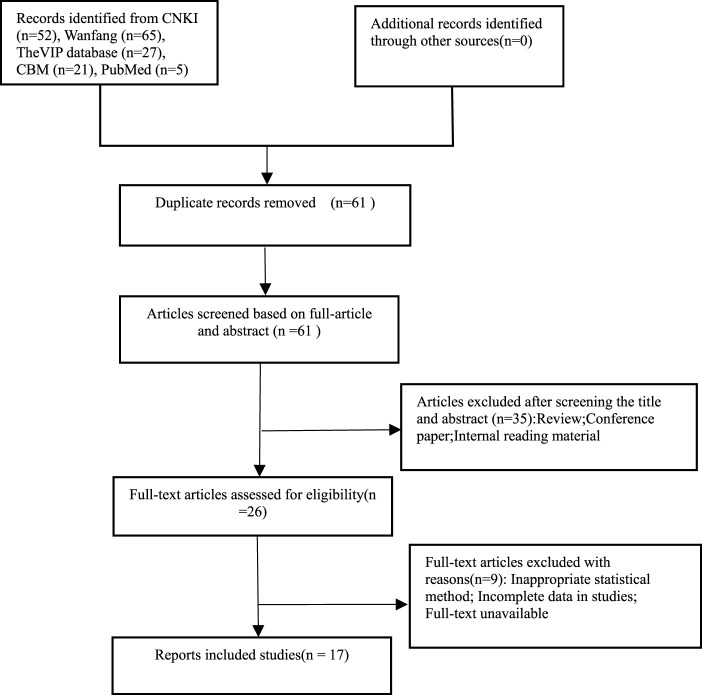
Literature screening process.

A total of 17 RCTs meeting the requirements were finally included in the study after removing duplicates and eliminating unqualified literature (Xu et al., 2021; Xu et al., 2019; Zhang et al., 2021; Tan and Zhu, 2023; Jang, 2023; Xia and Bao, 2022; Wu and Jia, 2022; Zhang et al., 2021; Chen et al., 2023; Li et al., 2023; Li and Li, 2023; Tong, 2022; Weng and Guo, 2022; Lu et al., 2022; Feng, 2023; Wang, 2022; Ouyang and Gui, 2022); the screening process is shown in Figure 1.

Seventeen clinical studies were included, with 2,778 cases (1,493 cases in the observation group and 1,285 cases in the control group). The minimum sample size of the observation group was 30 cases, and the maximum sample size was 599 cases. The observation group received edaravone dexborneol combined with basic treatment, whereas the control group received edaravone combined with basic treatment. Detailed characteristics of the included studies are presented in Table 1, whereas additional patient information is available in Supplementary Material S2.

**TABLE 1:** General information of included studies.

First author publication year	Sample size		Male/female		Age		Intervening measure		Course/d	Outcome indicator
	T	C	T	C	T	C	T	C		
Xu et al. (2021)	599	595	404/195	407/188	62.96	62.86	Edaravone dexborneol 37.5 mg, bid	Edaravone 30 mg, bid	14	①
Xu et al. (2019)	291	94	196/98	65/29	-	-	Edaravone dexborneol 12.5–62.5 mg, bid	Edaravone 30 mg, bid	14	①②⑤
Zhang et al. (2021)	40	40	12/18	19/21	62.5 ± 3.8	62.5 ± 3.8	Edaravone dexborneol 37.5 mg, bid	Edaravone 30 mg, bid	14	①②③④⑤
Tan and Zhu (2023)	39	34	22/17	20/14	63.1 ± 1.9	62.7 ± 2.4	Edaravone dexborneol 37.5 mg, bid	Edaravone 30 mg, bid	14	②④
Jang (2023)	38	38	22/16	22/16	52.6 ± 2.6	52.5 ± 2.6	Edaravone dexborneol 37.5 mg, bid	Edaravone 30 mg, bid	14	①②③④
Xia and Bao (2022)	46	46	-	-	-	-	Edaravone dexborneol 37.5 mg, bid	Edaravone 30 mg, bid	14	①②④⑤
Wu and Jia (2022)	49	49	28/21	29/20	67.8 ± 6.1	68.1 ± 6.6	Edaravone dexborneol 37.5 mg, bid	Edaravone 30 mg, bid	14	②③④⑤
Zhang et al. (2021)	30	30	17/13	16/14	63.5 ± 3.9	643 ± 4.2	Edaravone dexborneol 37.5 mg, bid	Edaravone 30 mg, bid	14	②④
Chen et al. (2023)	45	45	27/18	29/16	59.7 ± 7.5	60.3 ± 6.9	Edaravone dexborneol 37.5 mg, bid	Edaravone 30 mg, bid	14	①②③
Li et al. (2023)	53	52	26/27	27/25	58.0 ± 7.2	57.4 ± 7.0	Edaravone dexborneol 37.5 mg, bid	Edaravone 30 mg, bid	14	①⑤
Li et al. (2023)	40	40	22/18	24/16	55.1 ± 2.4	54.8 ± 2.3	Edaravone dexborneol 37.5 mg, bid	Edaravone 30 mg, bid	14	①③⑤
Tong (2022)	41	41	26/15	24/17	57.8 ± 4.3	57.7 ± 4.3	Edaravone dexborneol 37.5 mg, bid	Edaravone 30 mg, bid	14	①②④⑤
Weng and Guo (2022)	32	32	18/14	17/15	62.5 ± 3.1	62.4 ± 3.3	Edaravone dexborneol 37.5 mg, bid	Edaravone 30 mg, bid	14	①⑤
Lu et al. (2022)	45	45	29/16	26/19	63.0 ± 7.1	63.4 ± 7.8	Edaravone dexborneol 37.5 mg, bid	Edaravone 30 mg, bid	14	②③④⑤
Feng (2023)	30	30	13/17	15/15	66.3 ± 5.4	64.3 ± 2.3	Edaravone dexborneol 37.5 mg, bid	Edaravone 30 mg, bid	14	①②④⑤
Wang (2022)	44	44	25/19	22/22	65.4 ± 4.3	65.7 ± 4.6	Edaravone dexborneol 37.5 mg, bid	Edaravone 30 mg, bid	14	①②④⑤
Ouyang and Gui (2022)	31	30	15/16	16/14	60.8 ± 6.4	60.7 ± 6.5	Edaravone dexborneol 37.5 mg, bid	Edaravone 30 mg, bid	14	①②③⑤

Note: T, experimental group; C, control group; “-” means not mentioned; ① effective rate; ② NIHSS score; ③ Bl; ④ mRS; ⑤ adverse reaction.

### 3.2 Quality and risk assessment of included research methodologies

**FIGURE 2:**
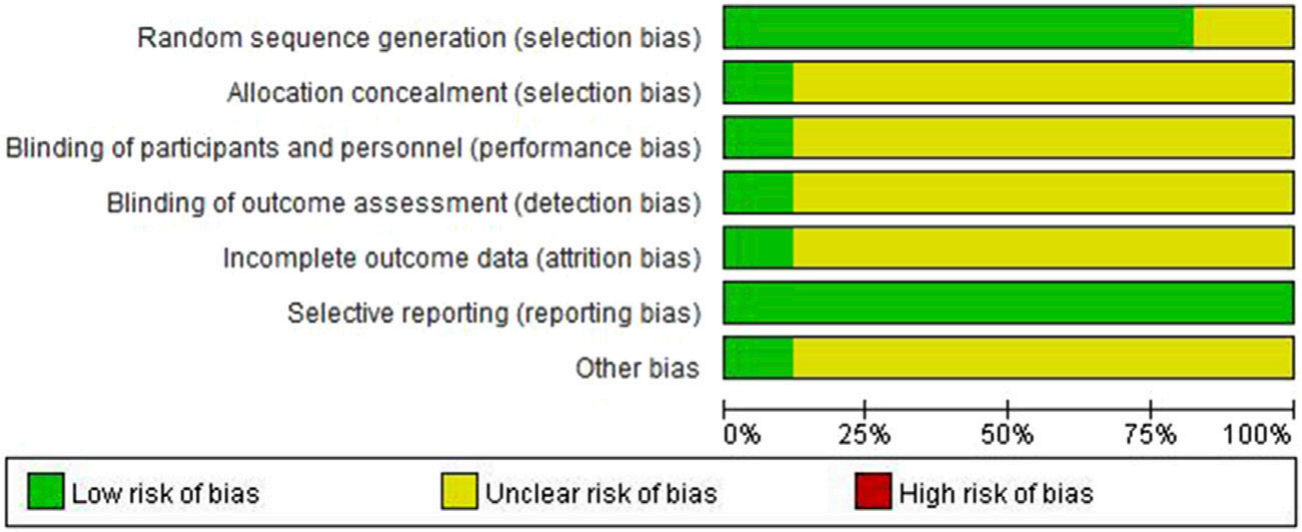
Risk of bias assessment.

Out of the 17 studies, 14 employed a randomization method. Specifically, 10 studies utilized the random number table method ((Xu et al., 2021; Xu et al., 2019; Zhang et al., 2021; Tan and Zhu, 2023; Xia and Bao, 2022; Chen et al., 2023; Li and Li, 2023; Tong, 2022; Lu et al., 2022; Wang, 2022), whereas four studies were grouped randomly (Jang, 2023; Zhang et al., 2021; Li et al., 2023; Feng, 2023). The remaining studies did not provide descriptions of their grouping methods (Wu and Jia, 2022; Weng and Guo, 2022; Ouyang and Gui, 2022). Only two studies implemented a double-blind design, and all data were reported in full, with no instances of loss to follow-up or attrition. Methodological quality evaluations are presented in Table 2. The risk of bias was assessed using RevMan 5.3 software, with the findings displayed in Figure 2.

**TABLE 2:** Methodological quality evaluation.

Literature resources	Random grouping method	Blind method	Shedding case	Literature quality
Xu et al. (2021)	Random number table	Double blind	Null	A
Xu et al. (2019)	Random number table	Double blind	Null	A
Zhang et al. (2021)	Random number table	Not mentioned	Null	B
Tan and Zhu (2023)	Random number table	Not mentioned	Null	B
Jang (2023)	Random allocation	Not mentioned	Null	B
Xia and Bao (2022)	Random number table	Not mentioned	Null	B
Wu and Jia (2022)	Not mentioned	Not mentioned	Null	B
Zhang et al. (2021)	Random allocation	Not mentioned	Null	B
Chen et al. (2023)	Random number table	Not mentioned	Null	B
Li and Li (2023)	Random allocation	Not mentioned	Null	B
Li and Li (2023)	Random number table	Not mentioned	Null	B
Tong (2022)	Random number table	Not mentioned	Null	B
Weng and Guo (2022)	Not mentioned	Not mentioned	Null	B
Lu et al. (2022)	Random number table	Not mentioned	Null	B
Feng (2023)	Random allocation	Not mentioned	Null	B
Wang (2022)	Random number table	Not mentioned	Null	B
Ouyang and Gui (2022)	Not mentioned	Not mentioned	Null	B

### 3.3 Meta-analysis results

#### 3.3.1 Total effective rate

**FIGURE 3:**
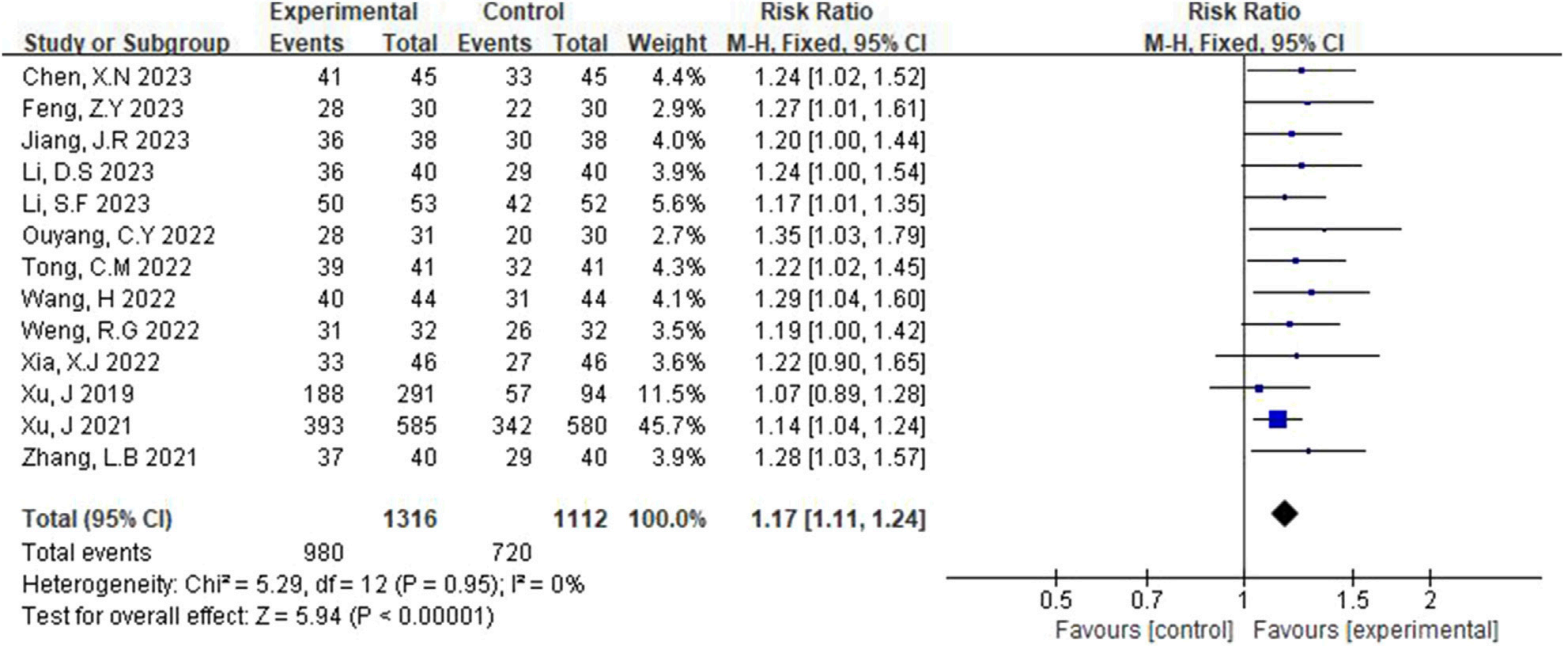
Forest plot of total effective rate.

Total response rates were reported in 13 studies (Xu et al., 2021; Xu et al., 2019; Zhang et al., 2021; Jang, 2023; Xia and Bao, 2022; Chen et al., 2023; Li et al., 2023; Li and Li, 2023; Tong, 2022; Weng and Guo, 2022; Feng, 2023; Wang, 2022; Ouyang and Gui, 2022), and fixed-effect models were used after heterogeneity was detected (p = 0.95, I^2^ = 0%). Figure 3 illustrates that the total effective rate in the experimental group was significantly higher than that in the control group, with a statistically significant difference (RR = 1.17, 95% CI [1.11, 1.24], p < 0.00001).

#### 3.3.2 NIHSS scores

**FIGURE 4:**
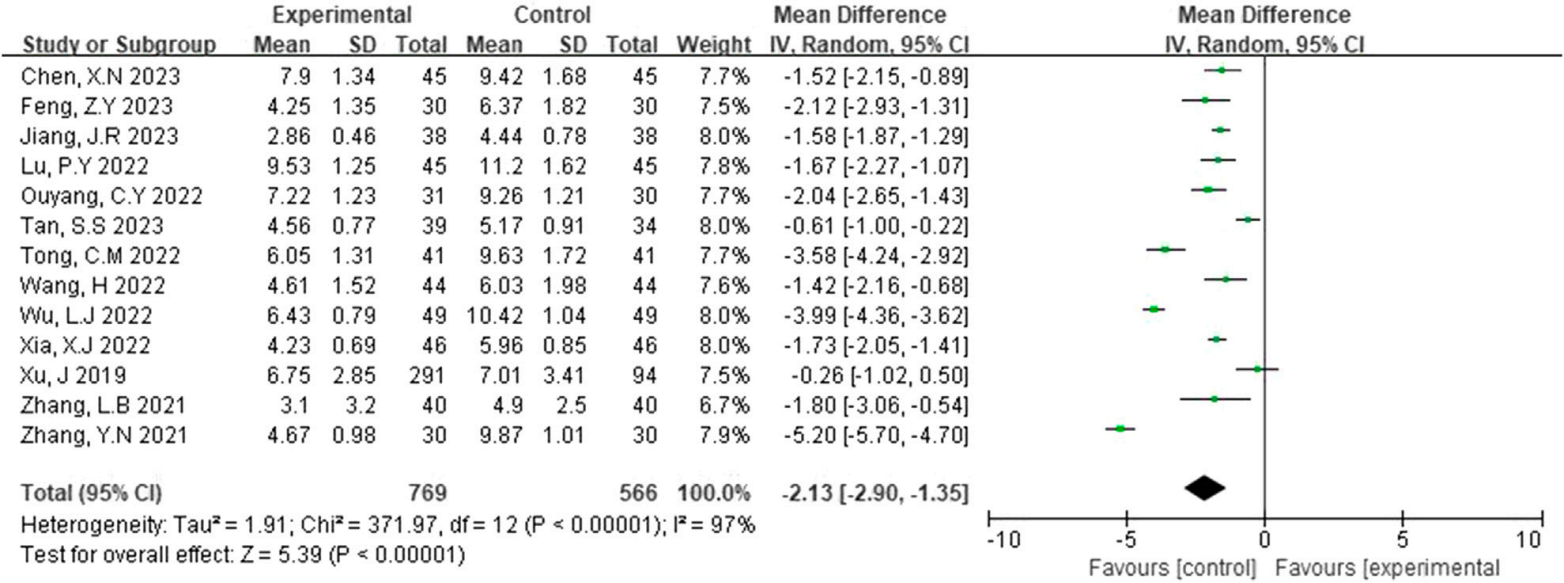
Forest plot of NIHSS scores.

NIHSS scores were reported in 13 studies (Xu et al., 2019; Zhang et al., 2021; Tan and Zhu, 2023; Jang, 2023; Xia and Bao, 2022; Wu and Jia, 2022; Zhang et al., 2021; Chen et al., 2023; Tong, 2022; Feng, 2023; Wang, 2022; Ouyang and Gui, 2022; Lu et al., 2022). A random-effects model was applied following the detection of heterogeneity (p < 0.00001, I^2^ = 97%). As shown in Figure 4, the NIHSS scores of the experimental group were significantly lower than those of the control group. This difference was statistically significant (MD = −2.13, 95% CI [-2.90, -1.35], p < 0.00001).

#### 3.3.3 BI

**FIGURE 5:**
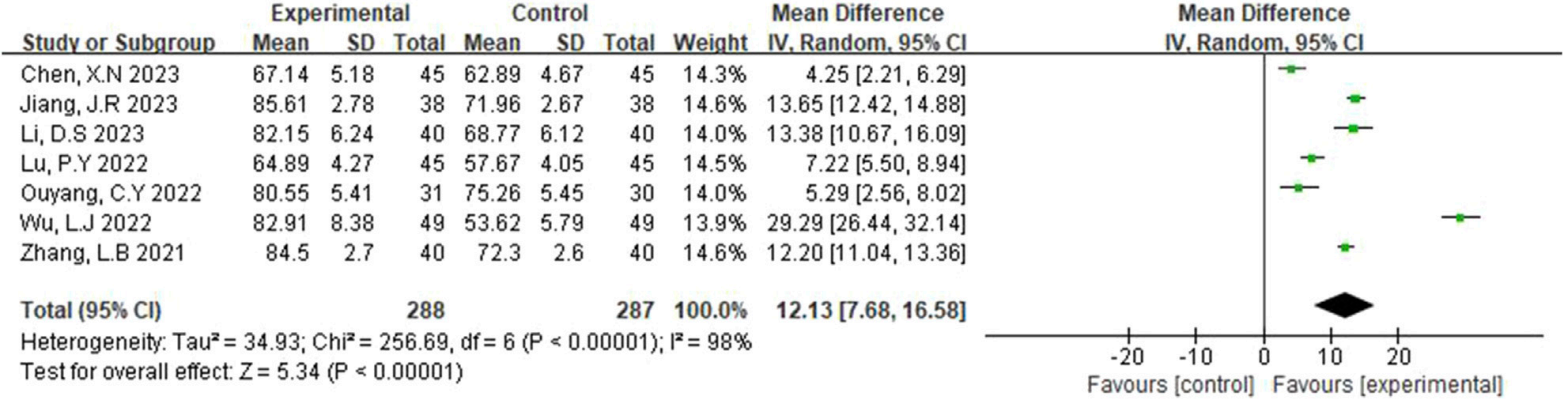
Forest plot of BI.

The Barthel Index (BI) was reported in seven studies (Zhang et al., 2021; Jang, 2023; Wu and Jia, 2022; Chen et al., 2023; Li and Li, 2023; Lu et al., 2022; Ouyang and Gui, 2022). A random-effects model was selected due to detected scores (p < 0.00001, I^2^ = 98%). As shown in Figure 5, the results of the systematic evaluation showed that BI in the experimental group was significantly higher than that in the control group (MD = 12.13, 95% CI [7.68, 16.58], p < 0.00001).

#### 3.3.4 mRS

**FIGURE 6:**
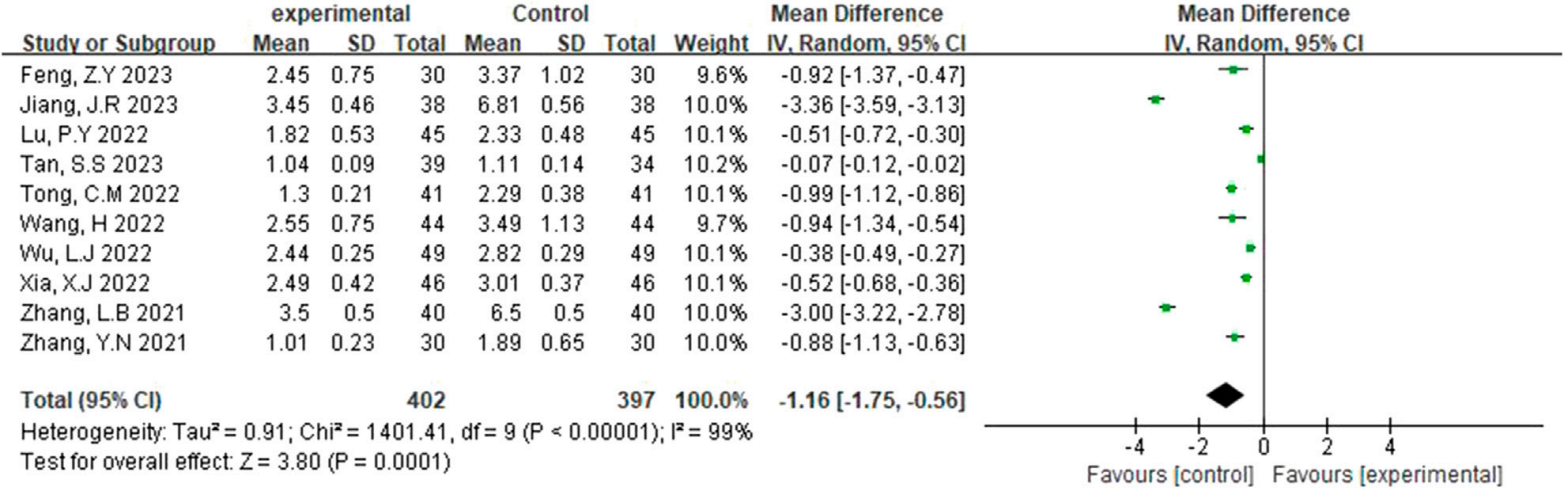
Forest plot of mRS.

The modified Rankin Scale (mRS) was reported in ten studies (Zhang et al., 2021; Tan and Zhu, 2023; Jang, 2023; Xia and Bao, 2022; Wu and Jia, 2022; Zhang et al., 2021; Tong, 2022; Lu et al., 2022; Feng, 2023; Wang, 2022), and a random-effects model was selected after heterogeneity (p < 0.00001, I^2^ = 99%). Figure 6 illustrates that the experimental group had a lower Rankin Scale score than the control group, with this difference being statistically significant (MD = −1.16, 95% CI [-1.75, −0.56], p = 0.0001).

#### 3.3.5 Adverse reaction analysis

Adverse reactions to edaravone dexborneol versus edaravone have been the focus of clinical research. In this study, the adverse reactions to edaravone dexborneol were analyzed in detail, and the results are as follows.

##### 3.3.5.1 Incidence of adverse reactions

**FIGURE 7:**
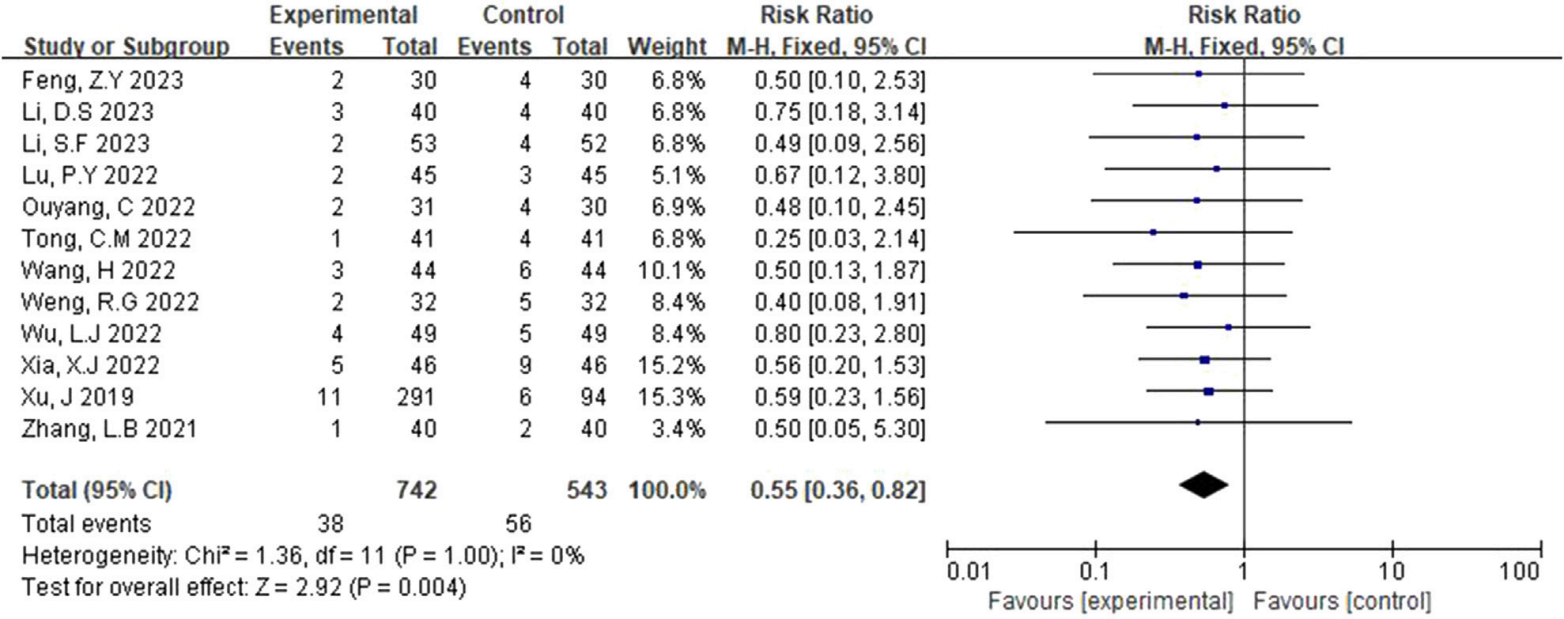
Forest plot of incidence of adverse reactions.

The adverse reactions rate was reported in 12 studies (Xu et al., 2019; Zhang et al., 2021; Xia and Bao, 2022; Wu and Jia, 2022; Li et al., 2023; Li and Li, 2023; Tong, 2022; Weng and Guo, 2022; Lu et al., 2022; Feng, 2023; Wang, 2022; Ouyang and Gui, 2022). The fixed-effects model was applied after heterogeneity was identified (p = 1.00, I^2^ = 0%). Figure 7 illustrates that the rate of adverse reactions in the experimental group was lower than that in the control group. The difference was statistically significant (RR = 0.55, 95% CI [0.36, 0.82], p = 0.004).

##### 3.3.5.2 Classification of adverse reactions

As shown in Table 3, the adverse reactions of edaravone dexborneol and edaravone mainly included bleeding (gingival bleeding, cerebral hemorrhage, and digestive tract bleeding), gastrointestinal reactions, and skin lesions (rash and skin irritation). Edaravone dexborneol showed fewer adverse and gastrointestinal reactions than edaravone. A subgroup meta-analysis of various adverse reactions was performed, and there was no statistical significance in the subgroup meta-analysis of bleeding, gastrointestinal reactions, or skin lesions (Table 4).

**TABLE 3:** Adverse reaction classification information (cases).

Literature resources	Gingival bleeding		Cerebral hemorrhage		Digestive tract bleeding		Gastrointestinal reactions		Skin lesions	
	T	C	T	C	T	C	T	C	T	C
Xu et al., 2019										
Zhang et al., 2021	1	1		1						
Xia and Bao 2022				1		1	3	4	2	1
Wu and Jia 2022							3	2	1	2
Li and Li 2023				1					1	1
Li and Li 2023										
Tong 2022							1	2		
Weng and Guo 2022				1		1	1	2	1	1
Lu et al., 2022							2	2	1	
Feng 2023				1			1	2	1	1
Wang 2022			1	1			1	2	1	3
Ouyang and Gui 2022				1					1	2
Total	1	1	1	7	0	2	12	16	9	11

**TABLE 4:** Subgroup analysis results of adverse reactions.

Type of adverse reaction	Literature quantity	Heterogeneity		RR (95% CI)	*P*
		P	*I* ^*2*^		
Bleeding	7	0.99	0	0.36 (0.12,1.04)	0.06
Gastrointestinal reactions	7	0.98	0	0.75 (0.36,1.56)	0.44
Skin lesions	8	0.95	0	0.82 (0.35,1.91)	0.65

### 3.4 Sensitivity analysis

**FIGURE 8:**
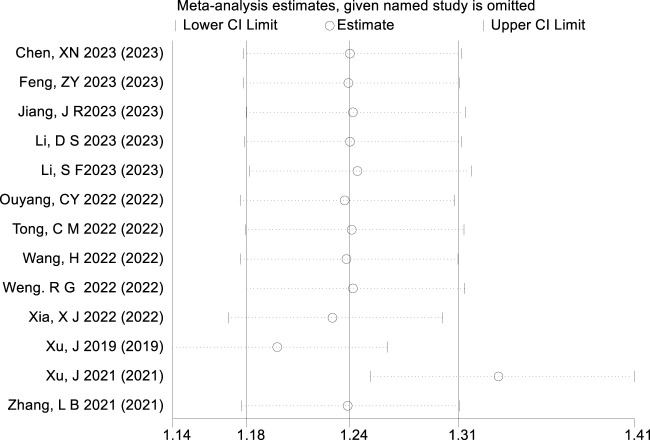
Result of sensitivity analysis.

The results of the sensitivity analysis conducted using Stata software are presented in Figure 8. When excluding the studies by Xu et al. (2021) and Xu et al. (2019), the point estimate of the combined effect size becomes significantly biased. Although the CI widens, it does not cross the null value, indicating that these two studies exert larger influence on the precision of the combined effect.

### 3.5 Publication bias analysis

**FIGURE 9:**
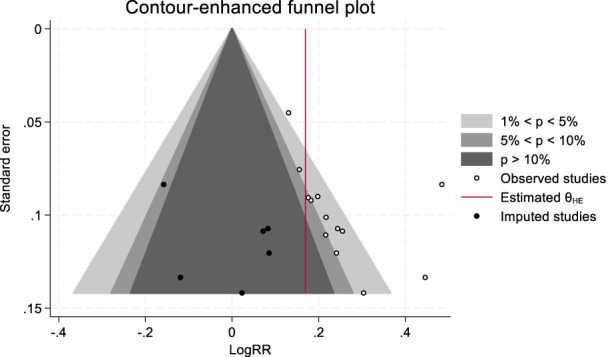
Trim and fill method of clinical efficacy.

This study examined publication bias using the trim and fill method. The analysis revealed that six potentially missing studies were filled, and these studies were all located in the statistically nonsignificant region (p > 0.05), suggesting that the original meta-analysis may have unpublished negative results, indicating the presence of publication bias (Figure 9). Despite the publication bias, the combined effect size adjusted by the trim and fill method remained in the original direction (LogRR = 0.221→0.169), and the 95% CI did not cross the null line, indicating the robustness of the results. Furthermore, the p-value obtained from the Egger regression test was less than 0.05, further supporting the potential presence of publication bias.

## 4 Discussion

Edaravone is a key neuroprotective agent for treating acute ischemic stroke, whereas dexborneol, a natural extract, protects brain nerves by reducing inflammation and excitotoxicity (Liu et al., 2011). The new drug, launched in 2020, is composed of edaravone and dexborneol in a 4:1 ratio. From a biochemical perspective, dexborneol is lipophilic and increases the brain levels of edaravone by approximately 30% by inhibiting the function of P-glycoprotein efflux pumps and enhancing the membrane fluidity of brain microvascular endothelial cells in combination therapy. From a pharmacological perspective, the two components work together to surpass the efficacy limitations of single-target drugs (Xu et al., 2021). This study evaluated 17 RCTs to determine the clinical effectiveness and safety of edaravone dexborneol in treating acute ischemic stroke. The findings revealed that edaravone dexborneol exhibited a more pronounced neuroprotective effect than edaravone alone. This was demonstrated by a higher overall effective rate and Barthel Index score among patients in the experimental group than among patients in the control group. Such results imply that edaravone dexborneol significantly improves patients’ functional status and quality of life. Moreover, the NIHSS and Rankin Scores in the experimental group were notably lower than those in the control group, reflecting enhancements in neurological deficits. Additionally, the experimental group encountered a reduced incidence of adverse reactions during treatment relative to the control group, with most reactions being mild gastrointestinal or dermatological issues, thereby indicating a favorable safety profile for edaravone dexborneol, which is encouraging for clinical use.

In addition, we conducted a sensitivity analysis and publication bias assessment on the overall effectiveness, and the results indicate that our findings are robust. The results indicate that our findings are robust. The Egger regression analysis and the trim and fill method revealed a potential risk of publication bias (p < 0.05). The trimmed pooled effect size remained stable, and the CI did not cross the null line. This suggests that the number or effect size of negative results is not enough to change our conclusions.

Overall, this study has several notable limitations: 1) most of the included studies have small sample sizes and poor methodological quality. 2) The limited number of studies included prevents comparisons of how factors such as age and gender affect outcome indicators. 3) Currently, the studies focus solely on the Chinese population, with no research conducted on other racial groups. 4) Some studies do not specify whether patients received reperfusion therapy prior to the study. Additionally, no subgroup analyses were performed regarding this aspect. 5) The short follow-up period limits the ability to analyze long-term effects. 6) The significant contribution of Xu et al.’s study (2021) (45.7% in the primary analysis) requires careful interpretation. The disproportionate weighting, primarily due to its larger sample size (n = 1,165 compared to a median of n = 41 in other trials), raises theoretical concerns about the potential overrepresentation of single-center evidence. Nevertheless, our sensitivity analyses demonstrated preserved significance upon exclusion, suggesting that the findings were not solely driven by this trial.

In summary, edaravone dexborneol has the potential to alleviate neurological deficits in patients with acute cerebral infarction, enhancing their daily living activities and mobility while improving clinical outcomes. Future multicenter trials with balanced sample sizes are needed to validate these observations.
